# Pre-treatment of Oil Palm Biomass for Fermentable Sugars Production

**DOI:** 10.3390/molecules23061381

**Published:** 2018-06-07

**Authors:** Nur Fatin Athirah Ahmad Rizal, Mohamad Faizal Ibrahim, Mohd Rafein Zakaria, Suraini Abd-Aziz, Phang Lai Yee, Mohd Ali Hassan

**Affiliations:** 1 Department of Bioprocess Technology, Faculty of Biotechnology and Biomolecular Sciences, Universiti Putra Malaysia, 43400 UPM Serdang, Selangor, Malaysia; nurfatinrizal@gmail.com (N.F.A.A.R.); mohdrafein@upm.edu.my (M.R.Z.); suraini@upm.edu.my (S.A.-A.); phanglaiyee@upm.edu.my (P.L.Y.); alihas@upm.edu.my (M.A.H.); 2 Laboratory of Biopolymer and Derivatives, Institute of Tropical Forestry and Forest Products, Universiti Putra Malaysia, 43400 UPM Serdang, Selangor, Malaysia

**Keywords:** lignocellulosic biomass, fermentation, pre-treatment, palm oil process flow

## Abstract

Malaysia is the second largest palm oil producer in the world and this industry generates more than 80 million tonnes of biomass every year. When considering the potential of this biomass to be used as a fermentation feedstock, many studies have been conducted to develop a complete process for sugar production. One of the essential processes is the pre-treatment to modify the lignocellulosic components by altering the structural arrangement and/or removing lignin component to expose the internal structure of cellulose and hemicellulose for cellulases to digest it into sugars. Each of the pre-treatment processes that were developed has their own advantages and disadvantages, which are reviewed in this study.

## 1. Introduction

Oil palm tree (*Elaeis guineensis jacq*.) was introduced to Malaysia by British in early 1870′s as an ornamental or decorative plant. The first commercial planting of oil palm was in 1917, in Tennamaran Estate in Selangor. The demand for oil during the industrial revolution in the 19th century as a lubricant in steam engines and machinery, and soap has led to the production of palm oil, which is also introduced to reduce the country’s economic dependence on rubber and tin [1]. Now, Malaysia is the second largest of world palm oil producers and exporters that accounts for more than 30% of world palm oil production and 37% of world exports in 2016 [2]. Being one of the biggest producers and exporters of crude palm oil (CPO) and palm oil products, Malaysia has an important role to fulfil the global need for oils and fats.

In line with the increase of the palm oil production capacity in Malaysia, a large amount of waste is being generated from this industry. Processing fresh fruit bunch (FFB) in the mill generates oil palm empty fruit bunch (OPEFB), oil palm mesocarp fiber (OPMF), oil palm kernel shell (OPKS), and a large amount of palm oil mill effluent (POME). Some biomass is also generated in the plantation area, such as oil palm frond (OPF) and oil palm trunk (OPT). In total, the palm oil industry generates more than 80 million tonnes of oil palm biomass in 2016, and the value will keep on increasing to fulfil the demand [3]. In order to maintain the sustainability of palm oil industry, a proper waste management has been developed and continuously improved to meet the economic and environmental challenges.

At the moment, the palm oil industries are still practicing the traditional waste management, with only little improvement. The oil palm biomasses, such as OPKS and OPMF, are still burned in a boiler to generate steam and electricity for mill operation [4]. The OPEFB is dumped at the mill without a proper treatment or brought to the plantation for mulching [5]. POME is still treated in ponds, whereby the final discharge still pollutes the environment, including changing the biodiversity of a nearby river [6]. Only a few factories have already implemented anaerobic digesters for treating POME and collecting methane [7]. Some factories, such as Global Green Synergy (http://www.ggs.my/) and Bionik Fertilizer (http://www.kulimnursery.com/), have started producing biocompost, biocharcoal, dried fiber, and pellets from oil palm biomass. Nevertheless, there is large potential for this biomass to be converted into various value-added products that will generate additional income to the industry, and at the same time, reduce the impact on the environment.

## 2. Oil Palm Biomass

**Figure 1:**
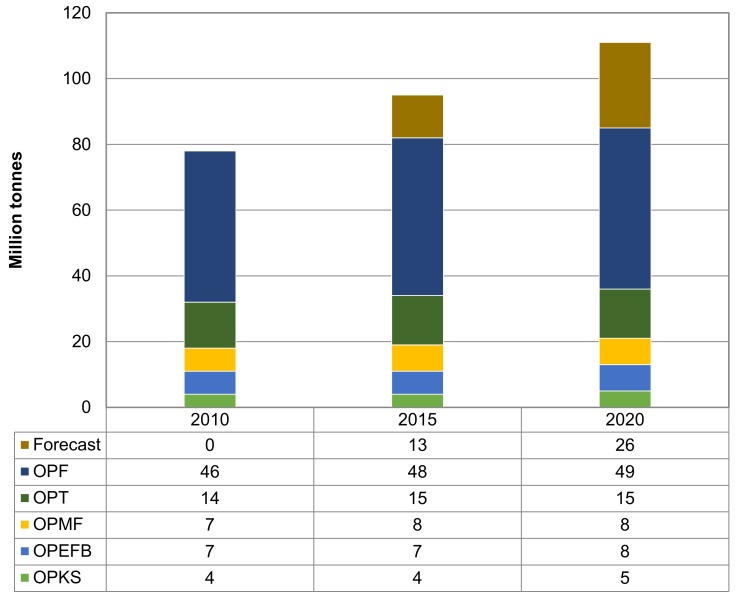
Production of oil palm biomass, i.e.,: oil palm frond (OPF), oil palm trunk (OPT), oil palm mesocarp fiber (OPMF), oil palm empty fruit bunch (OPEFB), and oil palm kernel shell (OPKS). Data obtained from Malaysia Innovation Agency [8].

Palm oil is the most important product for Malaysia that has helped to change the scenario of Malaysia agriculture and economy perspective. Palm oil industry provides a high economic return for Malaysia. The growth national income (GNI) is RM80 billion, which place this industry as the fourth GNI contributor in the country [8]. This industry becomes larger from year to year due to the world requirement on palm oil products. The plantation area for oil palm has gradually increased from 0.5 million hectares in early 1975 to 4.5 million hectares in late 2006 [9]. In 2015, Malaysia had 5.64 million hectares of oil palm plantations [10]. With the growth of palm oil industry, the amount of biomass residues generated also shows a significant increase. As a leading industry in the world’s oil production, the palm oil industry has left behind a large amount of biomass from its plantation and milling activities as compared to other types of agricultural biomass. Palm oil industry in Malaysia generates approximately 83 million tonnes of oil palm biomass in 2012 and it is expected to grow to be more than 100 million tonnes by 2020 [8]. Production of biomass from the Malaysia’s palm oil industry is shown in Figure 1.

**Figure 2:**
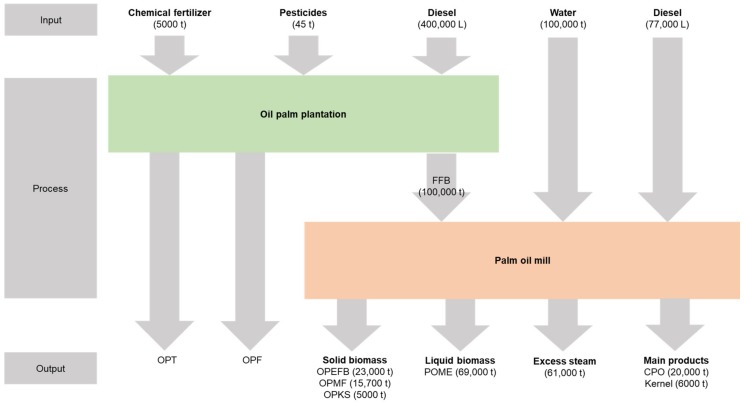
Material flow in the palm oil mill showing the production of oil palm empty fruit bunch (OPEFB), oil palm mesocarp fiber (OPMF), oil palm kernel shell (OPKS) and palm oil mill effluent (POME) from fresh fruit bunch (FFB). Units represent t: tonnes and L: litre. Data adapted from Yoshizaki et al. [13] and Hayashi [14].

The process flow of palm oil production at the mill with the production of oil palm biomass is illustrated in Figure 2. It shows that in every input of 100,000 tonnes of FFB for processing palm oil, a total of 43,700 tonnes of oil palm biomass residuals were generated. This value accounted for 23,000 tonnes of OPEFB, 5000 tonnes of OPKS, and 15,700 tonnes of OPMF. It should be noted that the OPEFB is the most abundant biomass produced from the palm oil mill. This value does not include the amount of biomass in wastewater, which is known as palm oil mill effluent (POME), which generates 69,000 tonnes per 100,000 tonnes of FFB input. In total, the palm oil mills in Malaysia generate 7.34 million tonnes of OPEFB, 7.72 million tonnes of OPMF, 4.46 million tonnes of OPKS, and 64 million tonnes of POME per year [3]. In the palm oil mill, only 10% of biomass is reused for electricity generation, while the remaining 90% are disposed of as wastes [11]. However, the occurrence of these oil palm waste has created a major disposal problem. The fundamental principles of waste management are to minimize and recycle the waste, recover the energy, and finally dispose of the waste [12]. 

Similar to other lignocellulosic biomass, oil palm biomass also consists of cellulose, hemicellulose, and lignin as major components of its cell wall, forming a fibre-like structure that makes the oil palm biomass recalcitrant. The cellulose and hemicellulose are polysaccharides that can be converted into sugar monomers, which can be used as fermentation substrates for various products. However, these components are protected by lignin, which is a complex and large complex structure containing cross-linked phenolic polymers that cover the internal layer of hemicellulose and cellulose [15]. It confers a rigid, impermeable resistance to microbial attack and oxidative stress [16]. The composition of lignin plays an important role in the selection of suitable lignocellulosic biomass as a substrate for sugar production prior to fermentation. A higher lignin content makes the structural arrangement rigid and highly ordered, therefore, it increases the biomass recalcitrance [17]. High lignin content will also contribute to the use of a vigorous pre-treatment process and/or require high amounts of enzymes or chemicals for delignification, subsequently increasing the total pre-treatment cost. Besides, the hydrolysis yield of sugar over the total biomass weight will also be less than for those substrates with a lower lignin content. 

In the case of oil palm biomass, OPF fibre showed the lowest composition of lignin. This fibre generated after being mechanically pressed to obtain sugar juice, leaving over the soft fibrous structure with lignin composition of less than 20%, as shown in Table 1. This biomass has been successfully pre-treated into sugar for various fermentation processes, such as biobutanol [18] and bioethanol [19,20]. However, the major challenge for utilizing this biomass as a fermentation substrate is the logistic approach to transfer the raw OPF to the processing plant since it is generated in the plantation area [21]. Another potential oil palm biomass is OPEFB. This biomass is the most abundant oil palm biomass that is produced in the mill that has been widely reported as a promising feedstock for sugar production. This is because this biomass is comprised of sugar of above 70% (cellulose + hemicellulose) and the lignin content is less than 25%, as shown in Table 1. Another abundant oil palm biomass that is produced in the mill is OPMF. However, a part of this biomass is burned in the boiler for steam and electricity generation in the mill. Besides, this biomass composed of 25–28% of lignin, higher than the lignin content presence in OPEFB. Studies have been conducted to pretreat OPMF to produce sugar [17,22,23]. 

Besides OPEFB and OPMF, palm oil mill also produces oil palm decanter cake (OPDC) and OPKS as waste. It should be noted that OPKS is fully utilized to generate steam and electricity by burning it in a boiler. However, several studies have been conducted to utilize this biomass in order to produce various value-added products. Most of OPKS is studied for biochar and activated carbon production, since this biomass is very compact, high density, and low moisture content, which are the criteria for biochar and activated carbon production [24,25,26,27,28]. It is also composed of very high lignin content of more than 50%, thus making this biomass unsuitable for sugar production. Another valuable oil palm biomass is OPDC, which is obtained from the three-phase decanter system. This oil separator functions to further extract the remaining oil present in POME before it is discharged as an effluent. The three-phase decanter extracts oil from sludge leaving over the remaining liquid (POME) and solid residue (OPDC) [29]. OPDC contains a significantly low amount of potential sugar of ~26% [29]. The major advantage of OPDC is that this biomass naturally has a small fibre size due to mechanical pressing during oil extraction [30]. Small particle size is also a key factor for the effective pre-treatment and hydrolysis by enzymes, since enzyme action is highly affected by the surface area [17]. Unfortunately, OPDC is not produced in all the mills in Malaysia since the three-phase oil separator is installed only in several palm oil mills. Besides, OPDC contains very high ash amount (22%) as it comes from waste mixed with water after the washing procedures. The lignin content is also considerably high at approximately 31%, which requires a harsh pre-treatment process to remove lignin. 

## 3. Pre-treatment

The structural arrangement of oil palm biomass and its composition play an important role for the conversion efficiency into fermentable sugars. In general, pre-treatment can be categorized into chemical, physical, physico-chemical, and biological pre-treatment [38], whereby each category has their own advantages and disadvantages, as shown in Table 2. Pre-treatment at upstream operation includes a physical pre-treatment, such as size reduction and thermo-chemical process that involve the disruption of the recalcitrant biomass. It increases substrate porosity by delignifying the lignin, hence, enables the maximal exposure of cellulose for cellulase action, which subsequently improve the hydrolysis process, minimize the energy consumption, and maximize the sugar recovery [39]. According to Taherzadeh and Karimi [40], an effective and economical pre-treatment should meet the following requirements: (1) avoiding destruction of hemicelluloses and cellulose, (2) avoiding formation of possible inhibitors, (3) minimizing the energy demand, (4) reducing the cost of size reduction for feedstock and cost of material for the construction of pre-treatment reactors, (5) consumption of little or no chemicals, and (6) using a compatible chemical. Effective pre-treatment is fundamental for optimal successful hydrolysis and downstream operations.

### 3.1. Physical Pre-treatment

Physical pre-treatment, also known as mechanical pre-treatment, is a process that uses mechanical methods, such as milling, chipping, grinding, and shredding to reduce the particle size and to increase the surface area of the biomass. Large biomass surface area increases the enzyme accessible area, and therefore increases the degree of depolymerization of biomass [44]. This pre-treatment is also able to partially modify the structure of biomass, reduce cellulose crystallinity, and disrupt the chemical bonding [36]. For example, the crystallinity index (CrI) of OPEFB reduced from 56% to 9% after grinding using ball milling for 120 min. A shorter milling duration is required for OPF, since this substrate has a lower lignin content than OPEFB [36]. In many practices, physical pre-treatment is used as an initial pre-treatment before the substrate is processed using other kinds of pre-treatment methods [40]. Sun and Cheng [42] reported that the selection of the pre-treatment method for chipping, grinding, and milling is depending on the final particle size of the biomass, usually in the range of 10–30 mm after chipping, and 0.2–2 mm after milling or grinding. Biomass size smaller than 400 mesh (0.04 mm) has little effect on the rate and yield of sugar production. In a study by Rizal et al. [17], the particle size was shown to play an important role in enhancing the hydrolysis of OPEFB and OPMF into glucose. The substrate with a size of 0.25 mm improved the hydrolysis yield by 4.6–4.8-fold. 

### 3.2. Chemical Pre-treatment

Chemical pre-treatment can be divided into two categories, which are acid and alkaline pre-treatments. The acid pre-treatment acts by solubilizing the hemicellulose fraction of biomass and exposes the cellulose to be converted to sugars [54]. It can be performed while using either concentrated or diluted acid. However, the utilization of concentrated acid is less desirable for the subsequent process due to the formation of inhibitors that inhibit enzyme action during hydrolysis and/or microorganisms during fermentation. Besides, its major disadvantages include serious corrosion problems and the use of sophisticated equipment for acid recovery, which leads to high operational and maintenance costs [55]. These limitations reduce the interest in applying this method on a commercial scale [56]. Examples of acid reagents used for pre-treatment process are hydrochloric acid (HCl), phosphoric acid (H_3_PO_4_), sulfuric acid (H_2_SO_4_), and nitric acid (HNO_3_) [12,25,26]. 

The alkaline pre-treatment involves the use of bases, such as sodium, potassium, calcium, and ammonium hydroxides [54,57]. This pre-treatment causes the swelling of lignocellulosic biomass, dissolution of lignin and hemicellulose, and de-esterification of intermolecular ester bonds, which subsequently reduces the extent of polymerization and CrI. Since alkaline pre-treatment dissolves mostly lignin, this pre-treatment is preferable than acids that dissolve carbohydrates. The disruption of lignin structure increases the exposure of the internal surface, and makes it accessible to enzyme digestion, which improves the hydrolysis efficiency [58]. The alkaline pre-treatment of OPEFB using 2% NaOH by Ibrahim et al. [59] produced approximately 32 g/L of sugar. Alkaline pre-treatment also has been tested on OPDC, which improved the sugar production from <1 g/L (untreated) to ~6 g/L after pre-treatment using 1% of NaOH [29]. A similar observation was also reported by Barlianti et al. [20] for the NaOH pre-treatment of OPEFB and OPF. 

Besides acids and alkaline chemicals, ionic liquids (ILs) and deep eutectic solvents (DES) are some of the chemicals that have been tested for the pre-treatment of lignocellulosic biomass. ILs are salts occurring in the liquid form that was employed to dissolve the lignocellulosic biomass [60]. Meanwhile, DES can be formed between a variety of quaternary ammonium salts and carboxylic acids capable of self-association, often through hydrogen bond interactions. This interaction forms a eutectic mixture with a melting point that was lower than that of each individual component [61,62]. ILs and DES is an inexpensive process, which can be conducted in low/mild temperature, non-toxic to enzymes or cells during saccharification and fermentation, and selectively depolymerize the lignin, thus making this pre-treatment process attractive to the industrial scale [60,63]. Besides, ILs and DES can also be recovered and recycled [60]. However, the recovery process requires a step to remove the inhibitor, in which the additional cost should be considered [45,46]. It was observed that approximately 11% of lignin composition was removed from OPF after being treated using ILs, which was higher than that of enzymatic delignification by laccase [63].

### 3.3. Physico-Chemical Pre-treatment

Physico-chemical pre-treatment involves both chemical and physical interactions in the pre-treatment process [64]. Steam explosion (SE), hydrothermal pre-treatment, ammonia fibre explosion (AFEX), liquid hot water (LHW), and supercritical CO_2_ (SC-CO_2_) are among the most widely used pre-treatment methods [55,65,66]. These pre-treatments are considered as the most effective, environment-friendly, and the process has been optimized with a variety of feedstocks on a pilot scale for industrial applications [31,44,67]. It has the capability of changing the structure of biomass, increasing the enzyme accessible surface area, and reducing the degree of biomass polymerization [38,44,68]. In addition, the modification of biomass structure using physico-chemical pre-treatment could enhance enzymatic hydrolysis [17,69].

Most of the physico-chemical pre-treatments are conducted at a high temperature and pressure, in aqueous solutions, and in a closed system [65]. The steam penetrates into the biomass and breaks the structural components, shearing the cell walls and partially hydrolyses the glycosidic bonds of hemicellulose. Physico-chemical pre-treatments use water to avoid the negative effects on the environment [70]. The effective pre-treatment mechanism occurs when autoionization of water at high temperature generates hydronium (H_3_O^+^) ions and reduces the pH, in which the solution formed acts, like an acid [67,70,71]. This acid is able to further solubilize the hemicellulose component and acetyl residues from xylan, which is then liberated in the form of acetic acid. This acetic acid will further catalyze the hydrolysis and this process is qualified as autohydrolysis [67]. Besides, it was reported that the concentration of hydronium ions from disassociation of acetic acid is higher than that from water autoionization [72]. 

Steam-assisted fractionation of lignocellulosic biomass improves enzymatic hydrolysis after an exposure to high pressure and temperature. After a certain pre-treatment duration, the biomass is rapidly decompressed, resulting in the degradation of lignocellulosic biomass and reducing its recalcitrance [31,73]. Typically, the oil palm biomass is treated at the temperature range of 160–240 °C and pressures of 20–50 bars for 1–120 min [22,49,73,74]. However, it should be noted that a higher temperature will cause severe degradation of cellulose, lowering the cellulose compositional percentage that will reduce the total glucose yield that was obtained after enzymatic hydrolysis. The selection of suitable temperature and pre-treatment duration is based on the types of oil palm biomass. It was reported that lower temperature is more efficient than a pre-treatment with higher temperature, even though at longer pre-treatment duration [57]. 

### 3.4. Biological Pre-treatment

Recently, this environmentally friendly approach has received renewed attention as a pre-treatment method for enhancing the enzymatic hydrolysis of lignocellulosic biomass into sugars. The major advantages of this pre-treatment are low capital costs, low energy usage, little or no chemicals involved, and can be conducted in mild environmental conditions [75,76]. Biological pre-treatment can be divided into two major categories, i.e.,: (1) microbial and (2) enzymatic pre-treatment. Microbial pre-treatment employs microorganism, including white fungi, brown fungi, soft rot fungi and bacteria to modify the lignocellulosic composition [52]. White rot fungi are most widely used for biological pre-treatment of lignocellulosic biomass as they effectively destroy the cell wall and lignin [77]. There are several types of white rot fungi such as *Phanerochaete chrysosporium*, *Clostridium butyricum*, *Trichoderma viride*, *Pycnoporus cinnarbarinus*, *Dichomitus squalens*, *Phlebia radiate*, *Trametes versicolor*, *Aspergillus oryza*, and *Pleurotus ostreaus* that have been investigated to pretreat different kinds of lignocellulosic biomass [66,67,78]. Lignin degradation by white rot fungi occurs through the action of lignin degrading peroxidases and laccase [78]. The major disadvantage of this pre-treatment is that it requires a long process duration to grow the fungi on lignocellulosic biomass.

Enzymatic pre-treatment employs enzymes to delignify the lignin component of oil palm biomass. The process is faster than microbial pre-treatment, as the enzyme will act directly on the biomass. The process takes about 72 h [79] as compared to a microbial pre-treatment that could take up to 40 days [80]. Enzymatic pre-treatment is also selective as it only attacks the lignin component, leaving the cellulose and hemicellulose intact. However, in order to effectively pretreat the biomass using enzymes, the biomass must first be mechanically pretreated to reduce the particle size as the substrate surface area affects the pre-treatment efficiency [17]. OPT was ground using wet disk milling to a size of 80 µm before being pretreated while using extracted enzymes that were produced by local isolates. They found that the mixture of enzymes rich of xylanase improves the hydrolysis efficiency of OPT [81]. The common ligninolytic enzymes are peroxidases, such as lignin peroxidase (LiP EC 1.11.1.14) and manganese peroxidase (MnP EC 1.11.1.13), as well as laccase (EC 1.10.3.2; benzenediol: oxygen oxidoreductase) [78]. All of these enzymes play a role in lignin degradation, and due to their dependence on molecular oxygen as opposed to hydrogen peroxide, they are becoming an item of interest in the industries related to enzyme [82].

### 3.5. Combination Pre-treatment

Combinations of different pre-treatments are always put into consideration in order to obtain an optimal fractionation of different components and achieve high yields of fermentable sugars. Selection of the pre-treatments to be combined and the sequence of the pre-treatment processes depend on the substrate characteristics, the availability of equipment, and the operational cost. The operational costs of pre-treatment are influenced by the pre-treatment duration, the amount of energy used, and the quantity of chemicals/enzymes that are applied in the process. For example, OPEFB and OPMF were ground using a hammer mill to a size of 0.25 mm, before being pretreated with laccase [17]. As an example, the OPF was ground to a size of 0.25–0.5 mm, before being pretreated using ILs, followed by an enzymatic delignification by laccase. This combination of pre-treatments resulted in a higher lignin removal as compared to single pre-treatment using either ILs or laccase [63]. OPEFB and OPF pretreated with hot compressed water (HCW) and wet disk (WD) milling has provided higher hydrolysis yield as compared to single pre-treatment [22]. A phosphoric acid pre-treatment, followed by fungal pre-treatment on OPEFB, reduced the CrI, which then improved the ethanol production in a simultaneous saccharification and fermentation (SSF) process [83]. 

A summary of the pre-treatment method from various studies is listed in Table 3. Different pre-treatment methods and conditions showed different yields of fermentable sugars obtained, which also contributed to different types of oil palm biomass used and the amount/composition of enzymes during enzymatic hydrolysis.

## 4. Conclusions

Although some pre-treatments could achieve up to 100% of glucose recovery, the efficiency and the suitability of the pre-treatment should be considered based on energy and time consumption, cost for chemicals and/or enzymes, initial capital for setting up the plant, the inhibitors released after the pre-treatment process, the waste generated from the pre-treatment process, the environmental impact, and the conversion of oil palm biomass into sugar. The mechanical pre-treatment to reduce substrate’s size is important since enzymatic hydrolysis by cellulase is highly affected by the accessible surface area to digest cellulose into glucose. Therefore, many mechanical pre-treatments, such as wet disk milling, hammer mill, and ball milling resulted in a high glucose recovery. Besides, even though a single pre-treatment could save the energy and time, a combination of more than two pre-treatments efficiently enhances the glucose or sugar recovery. However, the compatibility of combining the pre-treatments is limited, hence more research on the combination pre-treatment should be conducted.

## Figures and Tables

**Table 1:** Chemical composition of various raw oil palm biomasses

Oil Palm Biomass	Cellulose (%)	Hemicellulose (%)	Lignin (%)	Reference
Oil palm empty fruit bunch (OPEFB)	28–41	21–37	18–23	[17,31,32,33,34]
Oil palm mesocarp fibre (OPMF)	25–28	21–24	25–28	[17,35]
Oil palm kernel shell (OPKS)	28	22	44	[26]
Oil palm frond (OPF)	33	23	15	[36]
Oil palm trunk (OPT)	56	16	19	[37]
Oil palm decanter cake (OPDC)	22	4	31	[29]

**Table 2:** Advantages and disadvantages of various pre-treatment methods.

Pre-treatment Category	Pre-treatment Methods	Advantages	Disadvantages	References
Physical	Milling, grinding, chipping, shredding	• Easily reduces the substrate size and increases the surface area • Short process duration	• High energy requirement • High cost for equipment and machinery setup • Required additional pre-treatment as it does not remove lignin	[41,42]
Chemical	Acid	• Short process duration • Possible to fully hydrolyse the entire solid	• Corrosive on the equipment • Not economically feasible	[43,44]
	Alkali	• Short process duration • Selectively attacks the lignin	• Effective if the biomass has a low lignin content • Less effective if the lignin content is high in biomass • Usually requires high temperature to dissolve lignin	[44]
	Ionic liquids (Lis)	• Inexpensive • Not toxic to enzymes and fermentation • Can be recovered and recycled	• Inefficient on the recovery of ILs	[45,46]
Physico-chemical	Steam explosion	• No chemicals used • Low energy input • Environment-friendly	• Incomplete destruction of the lignin-carbohydrate matrix • Risk of condensation and precipitation of soluble lignin components • Destruction of a portion of xylan in hemicellulose • Inhibitor formed at higher temperatures	[44,47]
	Liquid hot water	• Hydrolysis of hemicellulose and removal of lignin • Lower temperature used • Fewer inhibitors produced at high temperature • Effective for large-scale application	• Amount of solubilised product is higher	[48]
	Superheated steam	• Improved energy efficiency • Low environmental impact when condensate is reused • Time saving • Environment friendly • Cost effective for large-scale	• Partial hemicelluloses degradation • Risk of condensation and precipitation of soluble lignin components • Not effective in removing lignin.	[22,23,49]
Biological	Fungi	• Selectively degrades lignin • Environment-friendly • Requires mild operating conditions	• Slow process and therefore it needs long process duration • Requires large space • Some carbohydrate fraction is consumed by the microorganism • Needs to be conducted in sterile conditions	[50,51]
	Ligninolytic enzymes	• Selectively degrades lignin and does not digest the carbohydrate structure • Environment-friendly • Requires mild operating conditions • Can be conducted in non-sterile conditions	• Enzyme cost is very high	[52,53]

**Table 3:** Pre-treatment performances on various oil palm biomass.

Pre-treatment Methods	Oil Palm Biomass	Pre-treatment Conditions	Yield	Reference
**Physical** Ball milling	Oil palm empty fruit bunch (OPEFB)	120 min	79% of glucose	[36]
**Physical** Ball milling	Oil palm frond (OPF)	60 min	84% of glucose	[36]
**Physico-chemical** Hydrothermal	Oil palm empty fruit bunch (OPEFB)	170–250 °C 10–20 min	100% of glucose	[73]
**Physico-chemical** Hot compresses water	Oil palm frond (OPF)	10 bar 178 °C 11.1 min 9.6 liquid-solid ratio	97% of glucose	[84]
**Chemical** Aqueous ammonia	Oil palm empty fruit bunch (OPEFB)	60 °C 12 h 21% of aqueous ammonia	41% of glucose	[85]
**Chemical** Solvent-ionic liquid	Oil palm frond (OPF)	80 °C 15 min 10% of solid loading	100% of glucose	[86]
**Chemical** Dilute acid pre-treatment at high temperature	Oil palm trunk (OPT)	3% H_2_SO_4_ 180 °C 40 min	80% of glucose	[87]
**Combination** Hammer mill Superheated steam (SHS) Laccase	Oil palm empty fruit bunch (OPEFB)	Size 0.25 mm SHS 160 °C, 20 min Laccase 100 U/g	72% of glucose	[17]
**Combination** Hammer mill Superheated steam (SHS) Laccase	Oil palm mesocarp fibre (OPMF)	Size 0.25 mm SHS 180 °C, 20 min Laccase 400 U/g	63% of glucose	[17]
**Combination** Alkaline hydrothermal and wet disk milling	Oil palm mesocarp fiber (OPMF)	1.5% NaOH	97% of glucose	[35]
